# Chronic Pulmonary Aspergillosis: Burden, Clinical Characteristics and Treatment Outcomes at a Large Australian Tertiary Hospital

**DOI:** 10.3390/jof8020110

**Published:** 2022-01-25

**Authors:** Olivier Despois, Sharon C-A. Chen, Nicole Gilroy, Michael Jones, Peter Wu, Justin Beardsley

**Affiliations:** 1 Faculty of Medicine and Health, The University of Sydney School of Medicine, Sydney, NSW 2145, Australia; Olivier.despois@health.nsw.gov.au (O.D.); sharon.chen@health.nsw.gov.au (S.C-A.C.); Nicky.Gilroy@health.nsw.gov.au (N.G.); 2 Department of Infectious Diseases, Westmead Hospital, Sydney, NSW 2145, Australia; 3 PRP Diagnostic Imaging, Sydney, NSW 2145, Australia; mwjones48@gmail.com; 4 Department of Respiratory and Sleep Medicine, Westmead Hospital, Sydney, NSW 2145, Australia; Peter.Wu@health.nsw.gov.au; 5 The Westmead Institute for Medical Research, Westmead, NSW 2145, Australia

**Keywords:** chronic pulmonary aspergillosis, chronic obstructive pulmonary disease, tuberculosis, mycoses, antifungal, *Aspergillus*

## Abstract

Chronic pulmonary aspergillosis (CPA) is a fungal lung infection associated with high morbidity and mortality. Yet, it remains under-recognized worldwide, with few Australian clinical data available. This retrospective study aimed to investigate CPA at a major tertiary referral hospital in Sydney. We identified patients having International Classification of Diseases (ICD-10) codes for “aspergillosis” and/or positive respiratory microbiology samples for *Aspergillus* species from January 2012–December 2018 at Westmead Hospital. Eligible cases were classified using European Respiratory Society 2016 CPA guidelines. We diagnosed 28 CPA patients: median age 60 years (IQR: 57–66), with 17 (60.7%) being males. Most had chronic cavitary pulmonary aspergillosis phenotype (n = 17, 60.7%). Twenty-three patients had outcomes data returned. Nineteen (82.6%) received antifungal therapy (median duration: 10.5 months (IQR: 6.5–20.7)). Eight (34.7%) patients received <6 months of antifungals, including three (38%) deaths. Two (13%) patients receiving ≥6 months of antifungals died. Chronic obstructive pulmonary disease (COPD) (n = 9, 32.1%) was the leading predisposing factor for CPA in our cohort. This contrasts with the global picture, where prior tuberculosis generally predominates, but is similar to findings from other high-income countries. Nevertheless, further larger-scale studies are required to determine whether these results are generalizable to the wider Australian population.

## 1. Introduction

Chronic pulmonary aspergillosis (CPA) encompasses a spectrum of progressive lung disease caused by infection with *Aspergillus* spp., most commonly *Aspergillus fumigatus* complex. It generally occurs in individuals with pre-existing structural lung defects or relatively minor immunosuppression secondary to comorbid disease [1,2].

Globally, CPA is estimated to affect over 3 million people and carries significant morbidity and mortality [2,3,4]. Prior pulmonary tuberculosis (TB) is the leading risk factor for developing CPA worldwide, although chronic obstructive pulmonary disease (COPD) appears to predominate in low TB-incidence countries [2]. 

CPA comprises five clinical phenotypes: chronic cavitary pulmonary aspergillosis (CCPA), subacute invasive aspergillosis (SAIA; formerly chronic necrotizing pulmonary aspergillosis (CNPA)), chronic fibrosing pulmonary aspergillosis (CFPA), *Aspergillus* nodule and simple aspergilloma, distinguished by radiological and clinical features [1]. The phenotype and severity of CPA is determined by dynamics of host–pathogen interactions [4]. 

CPA is under-recognized globally, and data on the CPA disease burden in Australia is lacking [2]. Consequently, awareness remains low amongst local practitioners [5,6]. Having a high index of clinical suspicion is important, as prompt diagnosis and initiation of antifungal therapy significantly improves CPA prognosis [7]. 

This retrospective single-center study provides insights into the predisposing factors, clinical characteristics and treatment outcomes of CPA patients in an Australian context.

## 2. Materials and Methods 

We identified potential patients (>18 years) for inclusion in this study by searching the Westmead Public Hospital inpatient electronic medical records, from 1 January 2012–31 December 2018, according to International Classification of Diseases (ICD-10) codes for aspergillosis (B44.0–B44.2, B44.7–B44.9 and J17.2), or positive microbiology, serology and/or histopathology for *Aspergillus*. Microbiology samples included a positive respiratory (sputum, bronchoalveolar lavage (BAL)) microbiology culture, BAL fluid Galactomannan (optical density intensity ≥ 0.5) or BAL polymerase-chain reaction result for *Aspergillus*. Serology included positive *Aspergillus* IgG (≥60 mgA/L) (cut-off value determined by local pathology laboratory protocols for the Phadia ImmunoCAP^™^ system), while histopathology involved visualization of *Aspergillus* fungal hyphae on lung tissue histology. 

We excluded cases with [7]:

- Allergic broncho-pulmonary aspergillosis (ABPA),
- Extrapulmonary aspergillosis,
- Clear alternative diagnoses,
- Severely immunocompromised (hematological malignancy, chemotherapy and transplant) patients.

Relevant clinical and radiological findings from remaining cases were transcribed onto pre-defined case report forms in REDCap [8,9]. These were subsequently reviewed by an experienced multi-disciplinary team (MDT), comprising 3 infectious disease and microbiology specialists, a respiratory physician and a pulmonary radiology specialist. We diagnosed CPA cases based on the 2016 European Society for Clinical Microbiology and Infectious Diseases/European Respiratory Society (ESCMID/ERS) CPA guidelines [7], which require all five of the following criteria to be met:

(1) Three months or more of pulmonary or constitutional symptoms (cough, sputum production, dyspnea, weight loss, hemoptysis), except in cases of SAIA where the clinical course is 1–3 months, or simple aspergilloma which may be asymptomatic,
(2) Serological/microbiological evidence of *Aspergillus* infection,
(3) Compatible radiological features,
(4) Exclusion of conditions which may mimic CPA (chronic cavitary pulmonary histoplasmosis, paracoccidioidomycosis, coccidioidomycosis and active pulmonary TB),
(5) Exclusion of major immunosuppressing conditions or current use of immunosuppressant medications (including chemotherapy and corticosteroid use >7.5 mg prednisone/day for >3 months). Value of corticosteroid use >7.5 mg/day for >3 months was arbitrarily set by the authors as being above the minimum threshold of acceptable immunosuppression for inclusion in this study.

Additionally, in this study the authors added a sixth diagnostic criteria:

(6) The presence of at least one marker of inflammation (white cell count (WCC) > 11.0 × 10^9^ cells/L, serum C-reactive protein (CRP) > 10 mg/L or elevated erythrocyte sedimentation rate (ESR) >30 mm/h) (stated WCC, CRP and ESR values were set as the cut-offs by the authors for inclusion in this study).

The MDT assigned CPA phenotype and primary predisposing factor by majority, requiring agreement of ≥3/5 specialists in an unblinded group setting. For consistency, we defined the date of diagnosis according to the earliest radiological evidence of CPA.

Treatment duration was calculated from initiation of antifungal therapy following radiological CPA diagnosis, until August 2019 (12 months from diagnosis of the last patient included in the cohort). Where antifungal dose was changed over the course of treatment, the dose on which the patient spent the longest time was recorded. Likewise, for patients treated sequentially with multiple antifungal agents, the total duration was recorded.

In line with previous published cohort studies, treatment outcomes were reported as overall ‘improved’ or ‘deteriorated’ by aggregating radiological and clinical outcomes at 6 and 12 months (±3 months) post-diagnosis [4,10]. Radiological improvement was defined as reduction in size/number of cavities and mycetomas, reduction in pleural and cavitary thickness or stability of radiological features on serial chest CT scans. Clinical improvement was defined as reduction in symptoms, as inferred from doctor’s clinic notes (self-reported patient symptom questionnaire reports were unavailable). In other words, overall improvement implies stability or improvement of the above parameters with lack of deterioration.

Deterioration was defined as a worsening in one or more of the above radiological and/or clinical features. Where radiological and clinical outcomes were discordant, we assigned the radiological outcome. In this study, we limited our outcomes analysis to the first 12 months following diagnosis.

Statistical analysis methods: Continuous data are presented as medians with interquartile range (IQR) and ranges. Categorical data are presented as number of individuals (%), unless otherwise specified. All analyses were performed using Microsoft Excel 2016, version 2102.

## 3. Results

**Figure 1:**
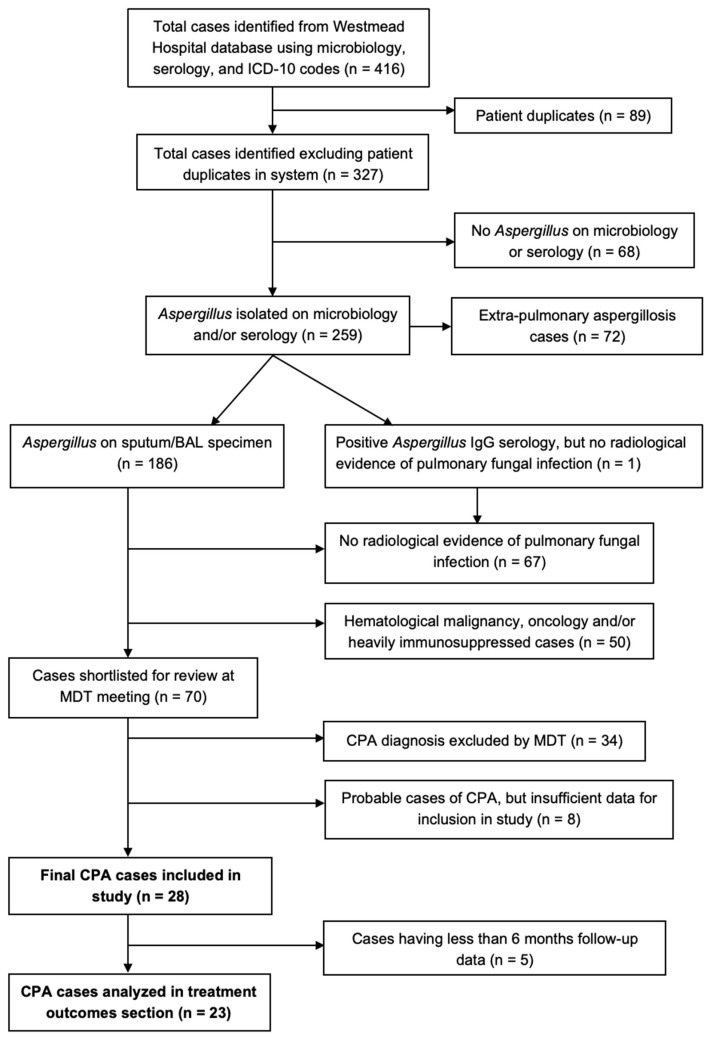
Flowchart showing cases included in study. Microbiology samples include sputum/broncho-alveolar lavage (BAL) fluid culture, BAL galactomannan, BAL polymerase chain reaction (PCR)-based tests or direct visualization of fungal hyphae on lung tissue histopathology. Serology included positive *Aspergillus* IgG results. MDT: multi-disciplinary team.

We identified 327 potential cases between 2012–2018. Twenty-eight were eventually diagnosed as having CPA. Of these, twenty-three had ≥ 6 months outcomes data available (Figure 1). 

### 3.1. General Characteristics

Table 1 shows our CPA cohort’s clinical characteristics. Median age was 60 years (IQR: 57–66; range: 19–83), with seventeen (60.7%) males. The most common predisposing factor for CPA was COPD (32.1%).

Table 1 also shows the most common CPA phenotype in our study was CCPA (60.7%). Three patients were diagnosed as having probable SAIA, in the absence of biopsy results, given their rapid clinical course (<3 months) [7]. 

CCPA: chronic cavitary pulmonary aspergillosis, SAIA: subacute invasive aspergillosis, CFPA: chronic fibrosing pulmonary aspergillosis, AN: Aspergillus nodule and SA: simple aspergilloma.

### 3.2. Clinical Symptoms

Clinical signs and symptoms at diagnosis in our cohort are shown in Table 2. The majority of patients (across all disease phenotypes) had respiratory symptoms of cough and sputum production, whilst constitutional symptoms of weight loss or chills/fever were less common. One patient with simple aspergilloma was asymptomatic (3.6%). 

### 3.3. Laboratory Examinations

Table 3 shows the laboratory results of our CPA cohort. Most patients had elevated inflammatory markers (CRP: 96.4%, n = 27; WCC: 60.7%, n = 17), and *Aspergillus* spp. isolated from a respiratory sample. Nine (32.1%) had positive fungal histopathology from lung tissue. Serology results (e.g., *Aspergillus* antigen/IgG, serum galactomannan) were not routinely available, hence were not assessed in this study. 

*A.fumigatus* complex was the most commonly isolated pathogen in our cohort (n = 20, 71.4%). One patient had an infection due to the *Aspergillus* *terreus* complex (3.6%) and another, the *Aspergillus* *niger* complex (3.6%). The *Aspergillus* species was unspecified in six cases (21.4%). 

### 3.4. Radiological Examinations

Table 4 shows the radiological features observed in our CPA cohort.

**Figure 2:**
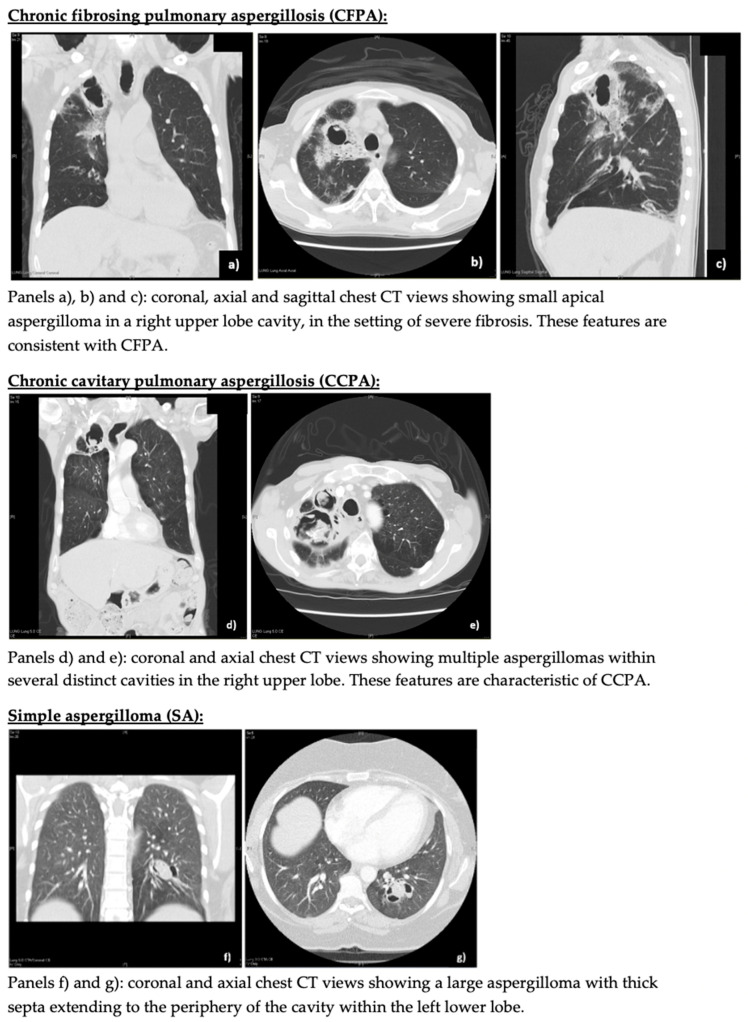
Representative CPA phenotype radiology.

Figure 2 shows some representative radiology.

### 3.5. Prognosis and Treatment Outcomes

Treatment outcomes were available for 23 patients (Table 5). Nineteen (82.6%) of these received antifungal treatment. 

Itraconazole was the most commonly administered antifungal agent (n = 15, 65.2%), given at a median dose of 100 mg BD (IQR: 100–200; range: 50–200). Eight (34.8%) patients received voriconazole (median dose: 200 mg BD (IQR: 200–200; range: 100–300)), and three (13.0%) posaconazole (median dose: 300 mg daily, (IQR: 250–300; range: 200–300)). Five (21.7%) patients changed antifungal agents due to side effects or poor response, while the remaining 14 (60.9%) continued on the initial antifungal agent.

Of the 23 patients in Table 5, fifteen (65.2%) were treated with antifungals for ≥6 months (median: 12 months, (IQR: 8–27; range: 6–44)), including two patients who underwent adjunctive surgical lung lobectomy (one CCPA and one SA patient). Four patients (17.4%) received <6 months of antifungals (median: 3 months, (IQR: 2–4; range: 1–3.5)). 

The remaining four (17.4%) patients received no antifungals: one underwent surgical excision for an *Aspergillus* nodule, while three others effectively received neither antifungal nor surgical treatment. The latter were either non-compliant with prescribed antifungal therapy or the diagnosis of CPA was originally missed, with patients instead treated for suspected infective exacerbation of ABPA/COPD. 

**Figure 3:**
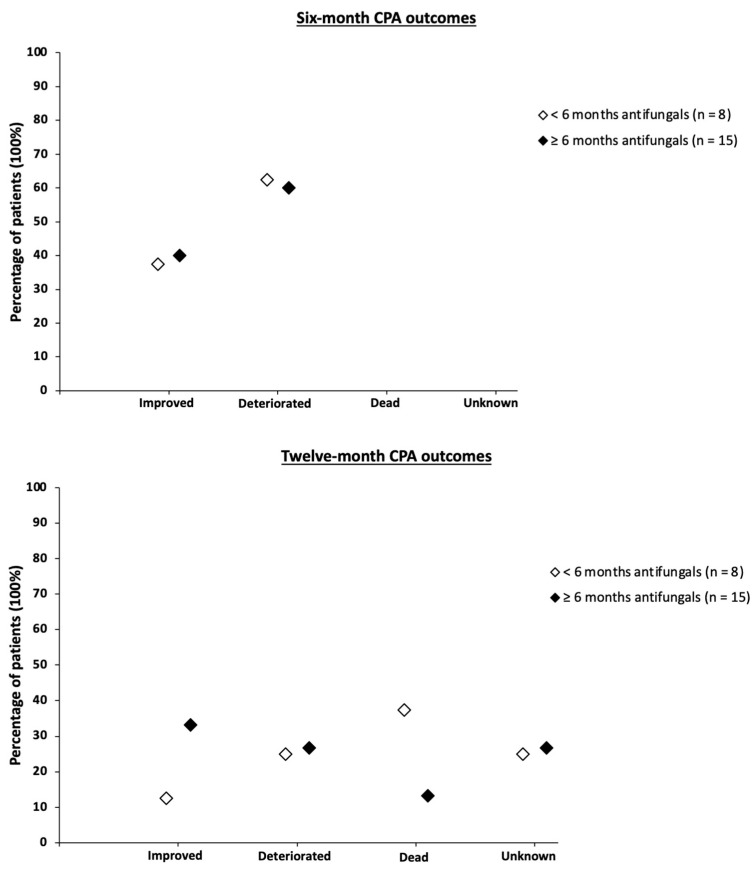
Treatment outcomes for CPA patients at six and twelve months post-diagnosis by antifungal treatment duration.

Figure 3 shows treatment outcomes for patients receiving long-term (≥6 months) antifungal therapy versus those receiving <6 months of antifungal therapy. 

N = 15 (filled diamonds) received ≥6 months of antifungal therapy. N = 8 (empty diamonds) received <6 months of antifungal therapy (including four patients who received no antifungals at all). CPA: chronic pulmonary aspergillosis.

Figure 3 shows that a similar proportion of patients had shown radiological/clinical improvement by 6 months between the long-term and <6-months therapy groups (40% and 37%, respectively). 

However, by 12 months, a comparatively greater proportion of patients on long term antifungals showed radiological/clinical improvement than patients in the <6-months therapy group (33% and 13%, respectively).

Overall, five patients in our cohort died, with a median delay of 9 months (IQR: 8–11; range: 6–11) between diagnosis and death. Three (38%) CPA patients in the <6-months antifungal therapy group were dead by 12 months post-diagnosis (median age at death: 72 years (range: 61–76)), compared to two (13%) in the long-term therapy group (median age at death: 60.5 years (range: 60–61)). All five of the deceased patients had the CCPA phenotype. The most common CPA predisposing factor among these patients was COPD (n = 3, 60%), with the other two patients having prior tuberculosis and mild immunosuppression due to alcoholism. Exact cause of death was not specified in the patient notes.

## 4. Discussion

As CPA is uncommon, it is challenging to identify a large cohort of patients. We conducted this study at Westmead Hospital, the principal tertiary referral hospital in Western Sydney, and identified 28 CPA patients within the 7-year study period [11]. This is comparable to previously published single-center case series from elsewhere, reporting 30–71 CPA cases over 10-year study periods [4,6,12].

A 2019 review article estimated the global CPA prevalence at 24 per 100,000 population, with rates being considerably lower in developed countries such as the UK, Portugal and Canada (5.7, 3.1 and 1.4 per 100,000 population, respectively) [13]. No Australian estimate was provided, though one might reasonably infer rates to be similar given prevailing socio-demographic trends in CPA burden [2,13]. Thus, Australian CPA prevalence likely lies between 1.5–6 per 100,000 population. Given our catchment population of approximately 1 million, one would expect 15–60 CPA cases within Westmead Hospital’s catchment area at any time [11]. Our present cohort size is towards the lower end of this estimate; thus, CPA likely remains under-diagnosed in our setting. 

Although, TB incidence is low in Australia, we hypothesized it would be the main underlying risk factor for our CPA cohort given the sizeable migrant community originating from high-TB incidence countries in Westmead Hospital’s catchment area [14]. The average local TB incidence over 2006–2015 in the Western Sydney Local Health District was 14.5 per 100,000 population—well above the New South Wales state average of 5.6 per 100,000 [14]. Overseas-born individuals from TB-endemic regions accounted for roughly 90% of TB cases in Western Sydney, with India, China, Sri Lanka and the Philippines being the most common countries of origin [14]. Nevertheless, chronic obstructive pulmonary disease (COPD) (32.1%) was the most common predisposing risk factor for CPA in our cohort, followed by prior pulmonary TB (17.9%) and mild iatrogenic immunosuppression (17.9%). This concurs with studies from other low-incidence TB countries in Western Europe, where COPD (29–47.2%) appears to overtake prior pulmonary TB as the leading risk for CPA [2,4,15,16,17].

Our results align with several case series, which reported their most commonly observed clinical symptoms were chronic cough (53–100%), sputum production (23.2–93%), dyspnea (7.2–68%) and weight loss (3–57%) [4,5,10,12,15,16,17,18]. Furthermore, one study reported 5.8% of their patients were asymptomatic, which is similar to our finding of 3.6% [5]. 

Our observed CPA phenotype frequencies were similar to previously published German (n = 71) and UK case series (n = 126), which reported CCPA (40.8–88.9%) as being most common [6,19]. However, our results contrast with findings of Chinese (n = 69) and French (n = 44) cohorts, which reported simple aspergilloma and SAIA as being most common, respectively [4,5]. The clinical importance of these differences is not clear, since CPA phenotypes likely reflect a spectrum of a single disease entity [1,2,7,19].

Most patients in our cohort were infected with the *A.* *fumigatus* complex (n = 20, 71.4%). This is expected as this species complex is the leading pathogenic species in most settings [2,13,20,21]. All patients had elevated inflammatory markers except for one with a histopathologically-proven simple aspergilloma, where an inflammatory response would be less likely [7]. 

We analyzed 23 patients’ treatment outcomes, including two (8.7%) who underwent surgical lobectomy prior to commencing long-term antifungal therapy. Similar adjunctive surgery rates (4–9%) have been reported elsewhere [3,10]. Surgical treatment is often reserved as a last resort, except in cases of easily resectable lesions such as simple aspergillomas, due to effects on residual lung function. Furthermore, surgery is associated with higher risks of morbidity and mortality in patients with severe, pre-existing cardiorespiratory comorbidities as may be the case with CPA patients [1]. Antifungal therapy is often indicated as a first line, with surgery being considered as an adjunct as appropriate, so it is unsurprising that only a handful of patients underwent surgery in this cohort [1].

The five patients for whom outcomes data were unavailable were either lost to follow-up, transferred to another facility/private clinic or they had their first follow-up at an interval longer than that analyzed in this study (i.e., >12 months).

Nineteen (65.2%) patients were treated with antifungals (median: 10.5 months (IQR: 6.5–20.7)). The most frequently administered antifungal was itraconazole (n = 15, 65.2%), followed by voriconazole (n = 8, 34.8%) and posaconazole (n = 3, 13%). Most patients (n = 14, 60.9%) remained on a single antifungal throughout their treatment, although five (21.7%) eventually changed antifungals due to side-effects. These findings are similar to those of a UK case series (n = 206), in which a minority (30%) of patients changed antifungals within the first 12 months, while most tolerated the initial agent [15]. In this study, all patients who changed antifungal agents were initially commenced on itraconazole tablets and only changed if they subsequently developed side-effects or they did not respond to the initial drug. The antifungal agents and median doses we describe are aligned with current therapeutic guidelines [7,20]. 

Patient outcomes at 6 months post-diagnosis were similar between the <6-months antifungal and long-term antifungal therapy groups (38% and 40% improved, respectively). However, at 12 months post-diagnosis a greater proportion (33%) of patients on long-term antifungals had improved compared to those in the <6-months antifungal group (13%). Furthermore, the one-year all-cause mortality was lower in the long-term antifungal group (13%) compared to the <6-months antifungal group (38%). We did not test for significance as our study was not powered to show any differences in outcome. The optimal duration of antifungal therapy in CPA is presently unknown, though current guidelines recommend at least 4–6 months of azole therapy for newly diagnosed CPA patients and our findings suggest longer treatment with antifungals improves prognosis [7]. 

The overall one-year all-cause mortality in this study was 21.7% (n = 5), based on twenty-three patients with outcomes data. This corresponds with previous case series reporting 6–80% CPA patient mortality in their cohorts [3,4,10,12,15,16,22,23]. Additionally, the observed median delay of 9 months between CPA diagnosis and death is comparable to a previously reported value of 12.4 months (IQR: 2.5–17.0) [10]. Although, the exact cause of death of patients in this study was unknown, the most common predisposing factor for CPA in this group was COPD (n = 3, 60%), with all five deceased patients having the CCPA phenotype. It is unclear whether this is because CCPA patients were disproportionately over-represented in our cohort or whether these patients had particularly severe underlying comorbidities.

The major limitation of our study is that, due to limited time and resources, we only searched for CPA cases using inpatient hospital admission databases. We would therefore have missed CPA patients seen in outpatient clinics, which could represent a significant proportion of all cases. Secondly, due to the retrospective design, we may be missing data results due to incomplete data sets. Thirdly, the delay from radiological diagnosis to initiation of antifungal therapy may occasionally be inflated since treatment is generally only commenced once patients become symptomatic. Furthermore, a longer follow-up period (> 12 months) would have been beneficial to assess the effect of antifungal treatment duration on patient outcomes in more depth.

Finally, *Aspergillus*-specific serological testing was carried out infrequently in our cohort, despite being part of current diagnostic guidelines for CPA [6,7,24,25,26], so we cannot comment on its value in this setting. It is not clear from our data whether the low frequency of serological testing is due to current clinical practice, or due to tests being ordered in private laboratories outside of the hospital system.

## 5. Conclusions

Given our hospital catchment area has a higher-than-average prevalence of tuberculosis relative to the rest of Sydney, we hypothesized that this would be the leading predisposing factor for CPA patients presenting to our hospital. However, COPD was found to be the most common predisposing factor among our patients. It is uncertain whether this observation is truly reflective of the wider Australian CPA population or if this is due to an incomplete capture of the CPA population in the area as a result of only searching inpatient admissions.

This is the first study to provide insights into the predisposing factors, clinical characteristics and burden of CPA in an Australian context. It is anticipated that this will stimulate further research and much-needed clinical studies in this field. Furthermore, our findings will help raise awareness of CPA amongst clinicians, especially those looking after patients with COPD, and will facilitate earlier diagnosis and management of patients with this condition. CPA should be considered in patients with a history of COPD, persistent cough and otherwise unexplained elevated white cell counts and CRP.

## 6. Future Directions

Future studies should utilize multi-site prospective data collection and outpatient encounters to characterize the Australian CPA prevalence and risk factors more accurately. 

## Figures and Tables

**Table 1:** Clinical characteristics of patients.

	Total (n = 28)	CCPA (n = 17)	SA (n = 4)	CNPA/SAIA (n = 3)	CFPA (n = 3)	AN (n = 1)
Median age (years), (IQR)	60 (57–66)	60 (58–65)	52 (38–68)	72 (50–74)	59 (58–59)	60 (NA)
Gender, n (%)						
Male	17 (60.7%)	12 (66.7%)	0	2 (66.7%)	2 (100%)	1 (100%)
Primary predisposing factor, n (%)						
Chronic obstructive pulmonary disease (COPD)	9 (32.1%)	7	0	1	0	1
Prior tuberculosis (TB)	5 (17.9%)	4	0	0	1	0
Mild iatrogenic immunosuppression	5 (17.9%)	1	1	2	1	0
Alcoholism	3 (10.7%)	2	1	0	0	0
Small airways disease	2 (7.1%)	1	1	0	0	0
Pulmonary interstitial fibrosis	1 (3.6%)	1	0	0	0	0
Diabetes	1 (3.6%)	0	1	0	0	0
Asthma	1 (3.6%)	1	0	0	0	0
ABPA	1 (3.6%)	1	0	0	0	0

When two or more predisposing factors were present, the primary one was included in this table. Mild iatrogenic immunosuppression-prolonged use of corticosteroids or rheumatoid biologics. CCPA: chronic cavitary pulmonary aspergillosis, SAIA: subacute invasive aspergillosis, CFPA: chronic fibrosing pulmonary aspergillosis, AN: *Aspergillus* nodule and SA: simple aspergilloma. ABPA: allergic bronchopulmonary aspergillosis. NA: not applicable.

**Table 2:** Clinical signs and symptoms by chronic pulmonary aspergillosis phenotype.

Symptoms Present	Total (n = 28)	CCPA (n = 17)	SA (n = 4)	CFPA (n = 3)	SAIA (n = 3)	AN (n = 1)
Cough, n (%)	22 (78.6%)	12 (70.6%)	3 (75%)	3 (100%)	3 (100%)	1 (100%)
Sputum production, n (%)	16 (57.1%)	10 (58.8%)	2 (50%)	1 (33.3%)	3 (100%)	0
Dyspnea, n (%)	13 (46.4%)	9 (52.9%)	1 (25%)	0	2 (66.7%)	1 (100%)
Weight loss, n (%)	6 (21.4%)	5 (29.4%)	0	0	1 (33.3%)	0
Hemoptysis, n (%)	2 (7.1%)	2 (11.8%)	0	0	0	0
Fever/chills, n (%)	2 (7.1%)	1 (5.9%)	0	0	1 (33.3%)	0
Asymptomatic, n (%)	1 (3.6%)	0	1 (25%)	0	0	0

Some patients had multiple clinical symptoms at diagnosis. CCPA: chronic cavitary pulmonary aspergillosis, SAIA: subacute invasive aspergillosis, CFPA: chronic fibrosing pulmonary aspergillosis, AN: *Aspergillus* nodule and SA: simple aspergilloma.

**Table 3:** Laboratory features by chronic pulmonary aspergillosis phenotype.

	Total (n = 28)	CCPA (n = 17)	SA (n = 4)	CFPA (n = 3)	SAIA (n = 3)	AN (n = 1)
Median WCC (×10^9^/L) (n = 17)	17.2	13.5	16.8	17.2	21.8	NA
(IQR)	(13.2–20.9)	(12.8–21.3)	(11.0–18.4)	(NA)	(20.9–22.7)	(NA)
(Range)	(5.2–27.6)	(11.7–27.6)	(5.2–20)	(NA)	(20–23.6)	(NA)
Median CRP (mg/L) (n = 27)	71	68.5	59	91	114	25
(IQR)	(33.5–139.2)	(44–182.2)	(37–178.5)	(51.5–107)	(71–127.5)	(NA)
(Range)	(12–298)	(22–287)	(15–298)	(12–123)	(28–141)	(NA)
Positive *Aspergillus* Sputum culture, n (%)	13 (46.4%)	9 (52.9%)	1 (25%)	0	3 (100%)	0
Positive *Aspergillus* BAL culture, n (%)	16 (57.1%)	11 (64.7%)	2 (50%)	1 (33.3%)	1 (33.3%)	1 (100%)
Positive *Aspergillus* BAL PCR, n (%)	15 (53.6%)	10 (58.8%)	2 (50%)	3 (100%)	0	0
Hyphae seen on histology, n (%)	9 (32.1%)	4 (23.5%)	3 (75%)	1 (33.3%)	0	1 (100%)

Laboratory data above were taken from the time of diagnosis (n = 24), or nearest available admission within 3 months of diagnosis (n = 4 patients). Lung tissue biopsy specimens were used for histopathology. CRP: C-reactive protein, WCC: white cell count, BAL: bronchoalveolar lavage, PCR: polymerase-chain reaction, NA: not applicable. CCPA: chronic cavitary pulmonary aspergillosis, SAIA: subacute invasive aspergillosis, CFPA: chronic fibrosing pulmonary aspergillosis, AN: *Aspergillus* nodule and SA: simple aspergilloma.

**Table 4:** Radiological characteristics by chronic pulmonary aspergillosis phenotype.

	Total (n = 28)	CCPA (n = 17)	SA (n = 4)	CFPA (n = 3)	SAIA (n = 3)	AN (n = 1)
Mycetoma/s, n (%)	25 (89.3%)	17 (100%)	4 (100%)	3 (100%)	1 (33.3%)	0
Consolidation, n (%)	20 (71.4%)	14 (82.3%)	0	3 (100%)	2 (66.7%)	1 (100%)
Pleural thickening, n (%)	19 (67.9%)	12 (70.6%)	1 (25%)	3 (100%)	2 (66.7%)	1 (100%)
Multiple cavities ± variable wall thickness, n (%)	16 (57.1%)	12 (70.6%)	0	2 (66.7%)	2 (66.7%)	0
Emphysema, n (%)	16 (57.1%)	11 (64.7%)	1 (25%)	2 (66.7%)	1 (33.3%)	1 (100%)
Bronchiectasis, n (%)	15 (53.6%)	9 (52.9%)	3 (75%)	3 (100%)	0	0
Fibrosis, n (%)	15 (53.6%)	11 (64.7%)	1 (25%)	2 (66.7%)	1 (33.3%)	0
Single cavity ± variable wall thickness, n (%)	11 (39.3%)	5 (29.4%)	4 (100%)	1 (33.3%)	1 (33.3%)	0
Bronchiolar nodules, n (%)	5 (17.9%)	5 (29.4%)	0	0	0	0
Halo sign, n (%)	2 (7.1%)	0	0	0	2 (66.7%)	0
*Aspergillus* nodule, n (%)	1 (3.6%)	0	0	0	0	1 (100%)

CCPA: Chronic cavitary pulmonary aspergillosis, SAIA: subacute invasive aspergillosis, CFPA: chronic fibrosing pulmonary aspergillosis, AN: *Aspergillus* nodule and SA: simple aspergilloma.

**Table 5:** Responses to antifungal therapy and outcomes for patients with ≥ 6 months follow-up (n = 23).

Antifungal Therapy	
Itraconazole	15 (65.2%)
Voriconazole	8 (34.8%)
Posaconazole	3 (13.0%)
No antifungal therapy	4 (17.4%)
Median delay to antifungal treatment (months)	0.7 (IQR: 0–8.1; Range: 0–33)
Median duration of antifungal treatment (months)	10.5 (IQR: 6.5–20.7; Range: 1–44)
Adjunctive surgical resection	2 (8.7%)
**Outcomes after treatment at 6 months**	
Improvement in symptoms	11 (47.8%)
Improvement in chest CT appearance	5 (21.7%)
Deterioration in symptoms	8 (34.8%)
Deterioration in chest CT appearance	11 (47.8%)
Dead	2 (8.7%)
**Outcomes after treatment at 12 months**	
Improvement in symptoms	5 (21.7%)
Improvement in chest CT appearance	3 (13.0%)
Deterioration in symptoms	7 (30.4%)
Deterioration in chest CT appearance	6 (26.1%)
Dead	5 (21.7%)

IQR: interquartile range.

## Data Availability

Data is available upon reasonable request from the authors.
